# The astounding exhaustiveness and speed of the Astral mass analyzer for highly complex samples is a quantum leap in the functional analysis of microbiomes

**DOI:** 10.1186/s40168-024-01766-4

**Published:** 2024-03-07

**Authors:** Thibaut Dumas, Roxana Martinez Pinna, Clément Lozano, Sonja Radau, Olivier Pible, Lucia Grenga, Jean Armengaud

**Affiliations:** 1 Département Médicaments Et Technologies Pour La Santé (DMTS), Université Paris-Saclay, CEA, INRAE, SPI, 30200 Bagnols-Sur-Cèze, France; 2 Thermo Fisher Scientific GmbH, 63303 Dreieich, Germany

**Keywords:** Tandem mass spectrometry, Microbiome, Proteotyping, Taxonomy, Functional analysis

## Abstract

**Background:**

By analyzing the proteins which are the workhorses of biological systems, metaproteomics allows us to list the taxa present in any microbiota, monitor their relative biomass, and characterize the functioning of complex biological systems.

**Results:**

Here, we present a new strategy for rapidly determining the microbial community structure of a given sample and designing a customized protein sequence database to optimally exploit extensive tandem mass spectrometry data. This approach leverages the capabilities of the first generation of Quadrupole Orbitrap mass spectrometer incorporating an asymmetric track lossless (Astral) analyzer, offering rapid MS/MS scan speed and sensitivity. We took advantage of data-dependent acquisition and data-independent acquisition strategies using a peptide extract from a human fecal sample spiked with precise amounts of peptides from two reference bacteria.

**Conclusions:**

Our approach, which combines both acquisition methods, proves to be time-efficient while processing extensive generic databases and massive datasets, achieving a coverage of more than 122,000 unique peptides and 38,000 protein groups within a 30-min DIA run. This marks a significant departure from current state-of-the-art metaproteomics methodologies, resulting in broader coverage of the metabolic pathways governing the biological system. In combination, our strategy and the Astral mass analyzer represent a quantum leap in the functional analysis of microbiomes.

Video Abstract

**Supplementary Information:**

The online version contains supplementary material available at 10.1186/s40168-024-01766-4.

## Background

Microbial communities are challenging biological systems due to the diversity of their components, their dynamics in time and space, intricate and redundant functional capabilities, and their myriad of possible interactions and networks. Microbiome research has seen many advances in establishing the nature of their components, pointing at key functionally relevant species, and predicting their functions based on metagenomics information [1]. By identifying proteins and monitoring their quantities, metaproteomics is a methodology that provides crucial information on the structural components, enzymes, and informational messengers of microorganisms, as well as on the host response, if any [2]. In addition to identifying the metabolic pathways in action and assessing their level of activity by means of their quantities, the methodology makes it possible to trace them back to the specific organisms that produced the corresponding proteins thanks to peptide sequences established by high-resolution tandem mass spectrometry. Metaproteomics therefore has a key role in deepening our knowledge of microbiomes, compared with methodologies limited to cataloguing microorganisms and genomic potential. Moreover, thanks to its extreme speed, this methodology could become an attractive new diagnostic tool for human medicine and the environment [3].

Microbiome research is strongly influenced by methodological advances. Recent developments in tandem mass spectrometry, acquisition strategies, and interpretation tools have great potential to transform metaproteomics into a high-performance methodology for deepening knowledge of microbial functioning. Metaproteomics grapples with an enormous amount of complex data, including giant databases of protein sequences built from metagenomic data or large numbers of sequenced organisms. Metaproteomics is also confronted with an exceptionally high number of proteins and variants from the sample, making the identification of common peptides easier than specific ones. Last, the lack of comprehensive coverage of the protein sequence database tends to decrease the outcome of the interpretation. Very recently, Stewart et al. [4] described the development of a new mass spectrometer that combines a powerful Orbitrap mass-resolving quadrupole, a novel ion processor rectilinear ion trap [5], and a revolutionary conceptual analyzer called Asymmetric Track Lossless (Astral) analyzer, enabling faster acquisition of high-resolution MS/MS spectra and high sensitivity compared with state-of-the-art mass spectrometers. The results demonstrated by this novel instrument for proteomics are promising in terms of depth of analysis with 10,000 groups identified from a HeLa peptide extract over a single 48-min run [4]. Such performance was further documented for comprehensive analysis of proteome post-translational modifications [6], plasma proteome [7], minimal cells [8], and single-cell proteomics [9]. Given these substantial improvements, in the present study, we explore its performance for profiling highly complex samples using a specific standard of human fecal material spiked with precise amounts of two bacterial proteomes. To fully exploit this new technology, we propose a novel workflow for metaproteomics, based on reliable proteotyping of microorganisms from short data-dependent acquisition (DDA), designing a specific database selecting the most valuable genomes, recording of high-density datasets in data-independent acquisition, and interpretation for increased coverage of the key players in the microbiota.

## Materials and methods

### MetaP reference sample

*Deinococcus proteolyticus* and* Balneola vulgaris* [10] were cultivated at 20°C with agitation at 140 rpm agitation in LB and Marine broth, respectively. Cells were harvested at the stationary phase by centrifugation. Human fecal material was obtained from a healthy adult donor. Proteins were extracted and proteolyzed into peptides with trypsin as previously described [11]. Peptides obtained from the two bacteria and the fecal material were quantified using the Pierce Quantitative Peptide Assays and Standards (Thermo Fisher Scientific) according to the manufacturer’s instructions, and then mixed at a ratio of 2:1:97 for *D. proteolyticus: B. vulgaris:* fecal material to obtain the MetaP reference sample.

### Orbitrap Astral mass spectrometry

NanoLC-MS/MS analysis was performed on an Orbitrap Astral MS coupled to a Vanquish™ Neo UHPLC system (Thermo Scientific™), interfaced with an EASY-Spray™ nano-source, and equipped with an IonOpticks-TS analytical column (25 cm × 75 µm) stabilized with a Heater THOR Controller (IonOpticks). The four gradients used were developed with 0.1% formic acid/99.9% H_2_O (Eluant A) and 0.1% formic acid/80% acetonitrile/19.9% H_2_0 (Eluant B): 8–35% B in 18 min followed by 35–45% B in 2 min (20 min gradient), 8–35% B in 25 min followed by 35–45% B in 5 min (30 min gradient), 8–35% B in 52 min followed by 35–45% B in 8 min (60 min gradient), and 3–17% B in 56 min followed by 17–25% B in 21 min and 25–34% in 12 min (90 min gradient), followed by a column wash at 95% B for 9 min and re-equilibration. Peptides (125 ng) were directly injected into the column. In DDA mode, the Orbitrap Astral MS was operated in positive mode with a fixed cycle time of 0.5 s with a full scan range of 400–1500 m/z at a resolution of 120,000. The automatic gain control (AGC) was set to “custom”, with a normalized AGC target of 300% and a maximum injection time of 50 ms. Precursor ion selection width was set at 2 Da. Peptide fragmentation was triggered by higher-energy collisional dissociation (HCD) with an HCD collision energy set at 30%. Fragment ion scans were recorded with the Astral analyzer with a scan range of 110–2000 m/z. In DDA mode, 30 min and 60 min gradients were tested in injection triplicates. In DIA mode, the Orbitrap Astral MS was programmed at the highest MS resolution (240,000) with a full scan range of 380 − 980 m/z. The normalized AGC target was set at 500%. For DIA measurements, the window width was set to 2 Da for the 15-min and 90-min gradients with a maximum injection time of 3 or 5 ms, respectively. This width was set at 3 Da for the 15 min, 30 min, and 60 min gradients with a maximum injection time of 3 ms, 7 ms, and 7 ms, respectively. The loop control function was activated (*N* = 100). The acquisition range was 150–2000 m/z after fragmentation of the isolated ions using HCD with 25% normalized collision energy (NCE). In DIA mode, 15 min, 30 min, 60 min, and 90 min gradients were tested in analytical triplicate. A quantity of 125 ng of peptides was injected per analytical run.

### Data interpretation for proteotyping organisms

Tandem mass spectrometry proteotyping was performed with each DDA dataset as previously described [12]. The top 100,000 MS/MS spectra were selected using Scanranker [13]. These MS/MS spectra were interpreted using Mascot version 2.6.1 (Matrix Science) against the NCBInrS database [12]. Peptide sequences were mapped to taxa at the species, genus, family, order, class, phylum, and superkingdom taxonomical ranks, as previously described [14], resulting in Taxon-to-Spectrum Matches (TSMs). TSMs and taxon-specific peptide sequences (spePEP) were used for the taxonomic identification of genera. Subsequently, a second round of search was initiated against a database derived from NCBInr encompassing all the identified genera and their descendants to identify the species.

### DB48 database creation

The most abundant species identified by proteotyping were used to create a specific-sample database. A total of 48 organisms were selected, and their annotated protein sequences were downloaded from NCBI, and merged in a single fasta file, resulting in the DB48 database, comprising 437,578 protein entries and totaling 169,873,349 amino acids. The DB48 spectral library for DIA interpretation was deposited in Figshare and is directly available for download (https://figshare.com/articles/dataset/DB48_SpectralLibrary_predicted_speclib/24638913).

### Data metaproteomics interpretation

The acquired DDA raw data file (60 min, replicate 3) was processed with Proteome Discoverer™ v3.1 software, using eventually SEQUEST™ with CHIMERYS™ search algorithms. Standard parameters were applied, with Carbamidomethylation of cysteines as fixed modification, Oxidation of methionines as variable modification, target FDR for PSMs and peptides of maximum 1%, minimum peptide length of 6, and FDR for proteins of 1%. DIA raw files were interpreted using DIA-NN 1.8.1 [15]. Deep learning-based spectral library generation was conducted in silico based on the DB48 database. A maximum of 2 missed cleavages were allowed, 2 variable modifications (oxidation of methionines and acetylation of the N-terminus), peptide length ranging from 7 to 30 residues, precursor charge of 2 and 3, m/z range from 400 to 1008, and fragment ion range from 200 to 1800 m/z. Automatic inference mode was selected for precursor and MS1 accuracy. Match between replicate runs and no shared spectra functions were activated. Protein inference was conducted based on protein names.

### Functional profiling of the host, microbiota, and spiked bacteria

The main protein within each identified protein group was employed for functional analysis, where the protein sequences were compared against the Kyoto Encyclopedia of Genes and Genomes (KEGG) database [16] using GhostKOALA tool [17]. KEGG orthologous (KO) terms were then assigned to KEGG pathways. In order to evaluate the depth of functional analysis, a percentage of pathway coverage was calculated by dividing the number of observed KO terms by the total number of KO terms in the pathway. Averaged protein abundance was used to quantify the weight of each pathway attributed to the host or microbiota. The contribution of each taxon within a function was illustrated using Circos tool [18]. The mapping of *Deinococcus proteolyticus* and *Balneola vulgaris* KO terms on the KEGG metabolic pathways was done with iPath 3.0 [19].

### Orbitrap Exploris 480 mass spectrometry and data processing

The metaP standard was analyzed in triplicates on an Orbitrap Exploris 480 (Thermo Scientific™) tandem mass spectrometer coupled to a Vanquish™ Neo pump module (Thermo Scientific™). Peptides were desalted on a reverse-phase PepMap 100 C18 μ-precolumn (5 mm, 100 Å, 300 mm i.d. × 5 mm, Thermo Scientific™) and separated on a 50-cm EasySpray column (75 mm, C18 1.9 mm, 100 Å, Thermo Scientific™) at a flow rate of 0.250 μL/min using a 90-min gradient (5–25% B from 0 to 85 min, and 25–40% B from 85 to 90 min) of mobile phase A (0.1% HCOOH/100% H_2_O) and phase B (0.1% HCOOH/100% CH_3_CN). DDA mode was activated with a full mass scan from 375 to 1500 m/z, an MS resolution of 120,000 and a MS/MS resolution of 15,000. Only peptides with 2 or 3 positive charges were selected for fragmentation with a dynamic exclusion time of 20 s and an isolation window of 0.7 m/z.

### Mass spectrometry proteomics data

Mass spectrometry proteomics data have been deposited to the ProteomeXchange Consortium via the PRIDE partner repository under the dataset identifiers PXD045838 (Orbitrap Astral DDA dataset), PXD046290 (15 and 30 min Orbitrap Astral DIA files), PXD046320 (60 and 90 min Orbitrap Astral DIA files), and PXD047139 (90 min Orbitrap Exploris 480 DDA files).

## Results

### DDA-based proteotyping to identify the most abundant organisms in the sample

**Fig. 1:**
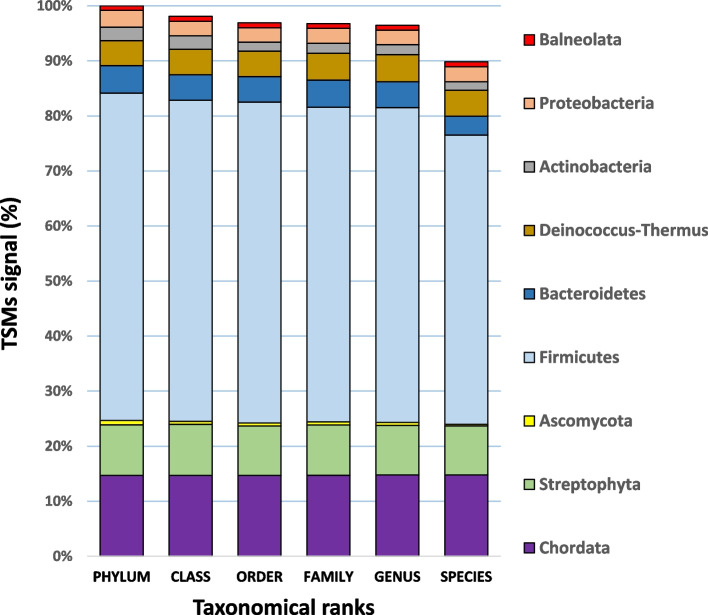
Relative proteotyping signals for different taxonomical ranks grouped by identified phylum. Protein biomass estimated based on the TSMs signal assigned to these microorganisms is grouped per phylum at various taxonomical ranks

NanoLC-MS/MS runs were carried out with 125 ng of the MetaP reference sample specifically created for this test of the Orbitrap Astral tandem mass spectrometer, using 30 min and 60 min gradients, in triplicate each. Proteotyping interpretation of these six datasets against a generic NCBInr-derived database encompassing protein sequence information from 50,995 different species was limited to the best 100,000 MS/MS spectra for each, as assessed by the Scanranker tool. This first round of searching was exploited to identify observable genera in each dataset. A database comprising all the descendants of the identified genera was built for each dataset and used to perform a second search to identify organisms at the species taxonomical rank. The species identified in these six independent analyses are listed in Table S1, along with the corresponding number of taxon-specific peptides and Taxon-to-Spectrum Matches (TSMs) at the various taxonomical ranks. Table S2 provides a reliable list of the 9 phyla, 44 genera, and 56 species that were proteotyped through the merging of these results, along with their respective contribution to protein biomass (Fig. 1). The overall TSMs signal decreases slightly when moving down the taxonomical hierarchy, from 100% at the phylum level to 96.8% at the family and 96.5% at the genus level, respectively. A more pronounced decrease is observed when moving from the genus to the species level (89.8%). This indicates that the genus level is well covered by the representative reference genomes in the database used for the proteotyping, while lower sequence coverage is observed at the species level. The proteotyping specificity of this taxonomical rank is therefore slightly lower compared to higher taxonomical ranks. Remarkably, for *Deinococcus proteolyticus, Balneola vulgaris,* and *Homo sapiens*, whose sequenced genomes are present in the database, no decrease is observed along the taxonomical ranks (Fig. 1). The largest decrease in the ratio is observed for Ascomycota and Actinobacteria, suggesting that the proteotyped species within these two phyla are only weakly representative. Given that their overall contributions are small, this has minimal impact on the results. In fact, the global TSMs signal, which represents 90% of the initial value, underscores the relevance of the species level across most phyla.

Amongst the 13 Eukaryota, the host *Homo sapiens* is logically the most abundant species, accounting for over 16% of the protein biomass. Eleven species affiliated to Streptophyta phylum were detected, representing a total of 9.9% of protein biomass, with *Glycine max*, *Helianthus annuus*, and *Oryza sativa* being the most abundant residual food components. Only one species assigned to Ascomycota, *Saccharomyces cerevisiae*, was identified. Among the 43 bacterial species identified, *Faecalibacterium prausnitzii*, *Anaerobutyricum halliii*, and *Coprococcus eutactus* were the most abundant, accounting for 8.2%, 7.3%, and 5.8% of protein biomass, respectively. The least abundant organism, *Clostridium bartlettii* CAG:1329, represented only 0.13% of the protein biomass but was reliably detected with 10 species-specific peptides in the best analytical run. No archaea were identified in this biological material sampled from a healthy young person on a meat-free diet. As expected, Firmicutes dominated the microflora with 33 identified species, accounting for 58% of the protein biomass. Notably, the two spiked bacteria never reported in the fecal microbiota, *Deinococcus proteolyticus* and *Balneola vulgaris,* were identified with 237 and 55 species-specific peptides in the best analytical run, respectively. They accounted for 5.22% and 1.01% of the protein biomass, respectively, as calculated from the TSMs signal assigned for the six interpreted datasets. While the second percentage corresponds very precisely to the expected added quantity of *Balneola vulgaris* (1%), the first value overevaluates the added quantity of *Deinococcus proteolyticus* (2%), indicating that the parsimony rules for establishing TSMs should be improved. The dynamic range of taxa assessed by metaproteomics shows that a relatively small number of genera dominate the sample in terms of biomass, with five genera (*Homo*, *Clostridium*, *Faecalibacterium*, *Coprococcus*, and *Anaerobutyricum*) contributing to almost 50% of the protein biomass. Next, we built the DB48 protein sequence database customized to represent only those organisms identified by tandem mass spectrometry proteotyping, which should account for the bulk of the protein biomass, i.e., 97.25% of the total (Table S3).

### Astral DDA current state-of-the-art metaproteomics is improved

**Fig. 2:**
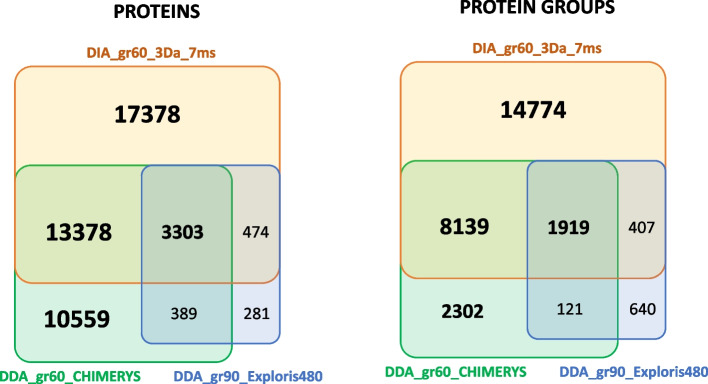
Venn diagram depicting the common and specific features of proteins and protein groups detected by three methodologies. The dataset used was acquired with a 60-min gradient (replicate 3) for chimerys DDA and DIA-NN DIA Orbitrap Astral, and 90 min gradient for Orbitrap Exploris 480

Due to the high diversity of peptides present in the MetaP standard, a large portion of the MS/MS spectra are expected to be chimeric, leading to a decrease in the assignment ratio. Preliminary testing was performed on a single 60-min DDA dataset (replicate 3) using the DB48 protein sequence database restricted to the most abundant organisms present in the sample. The new CHIMERYS algorithm (MSAID, Germany) integrated into Proteome Discoverer for the identification of chimeric spectra identified 158,716 PSMs, 42,996 peptide sequences, 27,628 proteins, and 12,480 protein groups. The ratio of MS/MS spectra assigned is 47.7% and the ratio of peptide sequences per protein group is 3.45, which are both rather high compared to most previously reported metaproteomics studies. This last interpretation demonstrates the high quality of the peptide preparation and proteolysis since 90.2% of the peptides have no missed cleavages, 9.5% of the peptides resulted from a unique missed cleavage, and only 0.3% are explained by two missed cleavages. In terms of precursor charge, 69.4% have 2 positive charges, 29.0% have 3 positive charges, and 1.6% have 4 charges. The differences in terms of assignation ratio and diversity of peptide sequence confirm the great benefit of artificial intelligence for interpreting very complex metaproteomics datasets. The Astral instrument performance was compared to the Orbitrap Exploris 480 using the exact same metaP reference sample. Each of the three 90-min DDA acquisition measurements generated an average of 94,751 (± 1.3%) MS/MS spectra, resulting after querying the DB48 in an average of 16,552 (± 3.2%) PSMs, 12,749 (± 2.3%) peptide sequences, and 3628 (± 0.9%) protein groups (Table S4). Those results are consistent with those reported for other human fecal samples using the same instrument but with a longer gradient [20]. In DDA mode, the performance of the Astral and CHIMERYS tool interpretation resulted in almost 10 times more PSMs, a threefold increase in the number of peptides and protein groups identified, with only two-thirds of the dedicated time to mass spectrometry. Figure 2 shows the comparison between the two instruments in terms of protein coverage. These results indicate the new current state-of-the-art metaproteomics that can be achieved based on DDA datasets acquired with the Orbitrap Astral tandem mass spectrometer.

### DIA metaproteomics increases significantly peptide coverage and protein detection

**Fig. 3:**
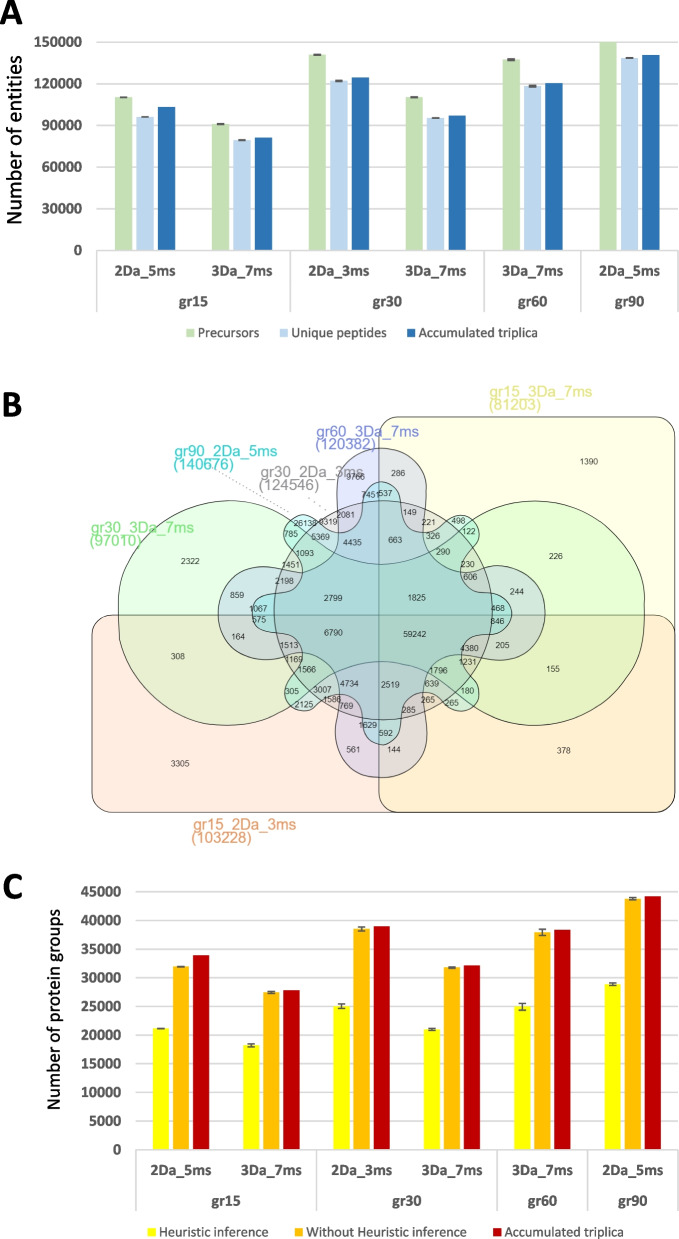
DIA interpretation results for the five conditions tested in triplicate.** A** Average numbers of detected precursors and unique peptides for the three replicates, and accumulated unique peptides. **B** Venn diagram of unique peptides among the five conditions. **C** Average numbers of protein groups with or without heuristic inference, and accumulated protein groups when summing the three replicates

To investigate the Astral performance in DIA mode and assess the extent to which it improves the depth of metaproteome knowledge, DIA analyses with different LC–MS/MS acquisition parameters were carried out. We recorded triplicate analyses of the MetaP standard using gradients spanning 15, 30, 60, and 90 min. Data interpretation was performed with a spectral library generated in silico for DIA-NN, and the results were presented either in normal mode or in heuristic protein inference mode. This last option avoids protein accession redundancies across multiple protein groups, giving more appropriate results for subsequent functional analysis, albeit with a reduction in the number of protein groups listed. This data interpretation shows improved interpretation compared to the DDA dataset (Fig. 2) and high reproducibility between replicates (Fig. 3). A total of 188,442 peptide sequences are observed when totaling the 18 analytical runs and 59,242 peptide sequences are observed across all six conditions. For example, in the dataset acquired in 30 min of gradient (2 Da fragmentation window and 3 s injection time), 140,857 (± 396) precursors, 122,087 (± 346) peptide sequences, and 38,528 (± 58) protein groups were observed in average per analytical run. When cumulating the three replicates, a total of 124,546 peptide sequences and 38,987 protein groups were observed under these experimental conditions. Logically, the longer gradient tested, 90 min, resulted in a larger landscape with an average of 138,596 unique peptides and 44,204 protein groups. For the 60-min gradient, 118,262 peptide sequences and 37,934 protein groups were observed, thus a significant increase compared to the 60-min gradient DDA CHIMERYS analysis with × 2.7 and × 2.4 fold change, respectively. Overall, these results emphasize the benefit of DIA analyses for peptide and protein identification. Noteworthy, the 15 min gradient (2 Da–5 ms) performs very well with 96,102 peptide sequences identified and 31,928 protein groups, with an average of 3.0 peptides per protein group.

### Significantly improved panorama of metabolic pathways relies on extra-large DIA peptidome landscape

To unveil the full potential of the Orbitrap Astral mass spectrometer to advance our understanding of diverse biological systems, we further explored the results of the 30-min DIA gradient (2 Da–3 ms), as it offers a good compromise between acquisition time and performance. The proteins identified were annotated using the KEGG database. Across the three analytical replicates, a total of 25,283 distinct protein groups were successfully annotated, with an average of 25,019 ± 33 protein groups per analytical run. Of these, 997 proteins originated from the host (18.1% of cumulated abundance), 2,036 proteins from the residual diet (8.2% of abundance), 20,418 from the microbiota (69.3% of abundance), while *B. vulgaris* and *D. proteolyticus* accounted for 743 and 1089 proteins, 1.1 and 3.4% of abundance, respectively (Table S5). It should be noted that the latter two percentages are in relatively good agreement with the experimental design of the MetaP sample, indicating that cumulative protein abundance could be a good indicator for estimating the percentage biomass of each taxon. This also shows that the interpretation of the DIA and DDA datasets is fairly convergent when it comes to establishing the percentage biomass of a taxonomic unit, although here two different surrogate variables, namely protein intensity and TSM, were used.

**Fig. 4:**
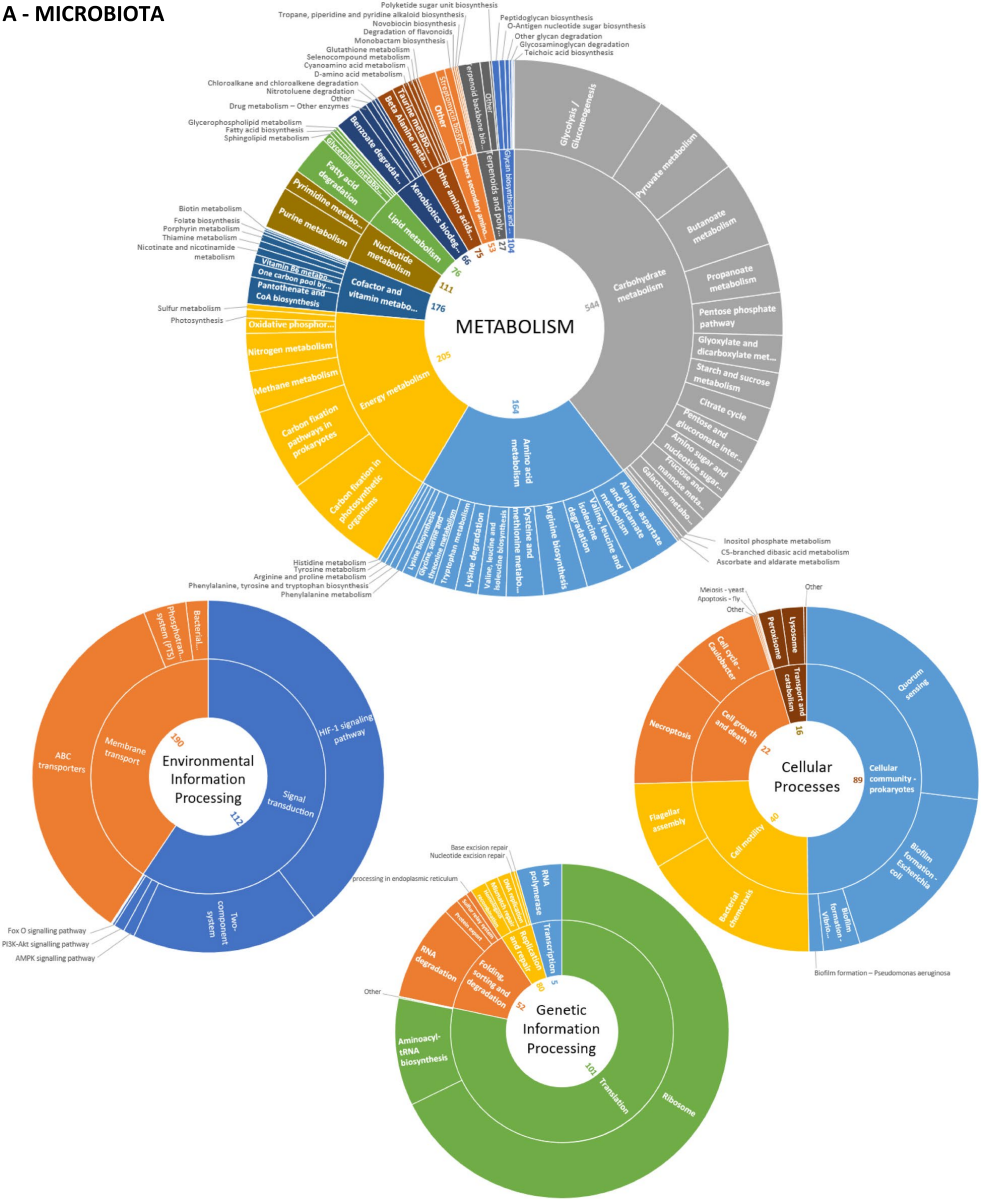

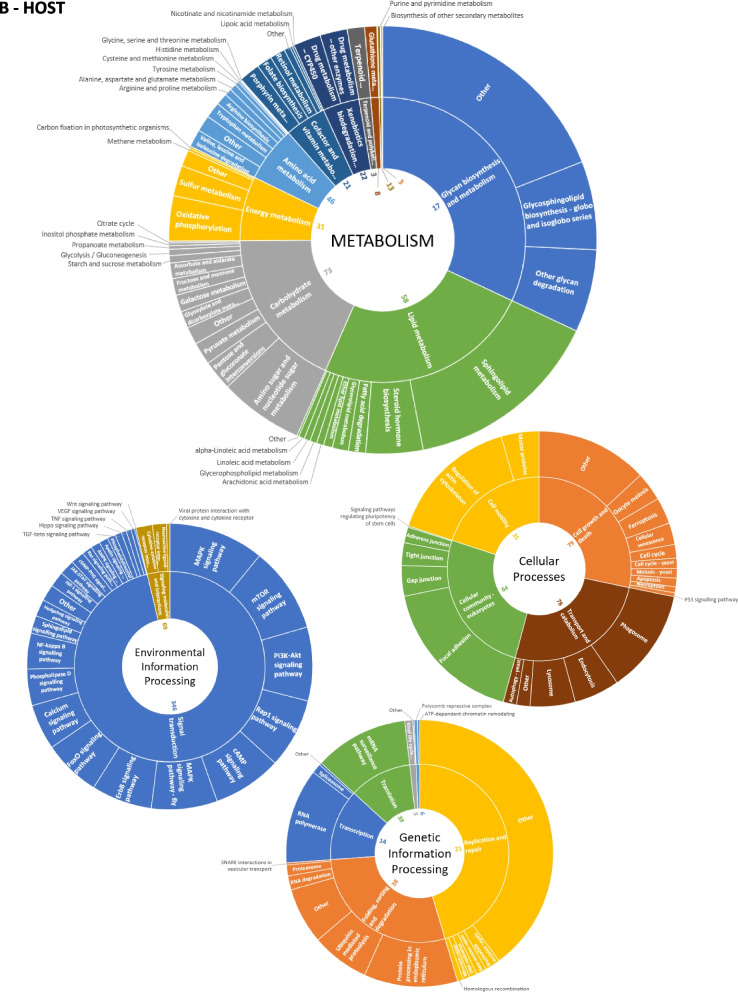
Abundance of all identified KEGG pathways for the host and the microbiota.** A **Host. **B **Microbiota. The proportion of the pathway depends on the protein biomass. The inner numbers represent the number of KO terms recorded in the pathways

**Fig. 5:**
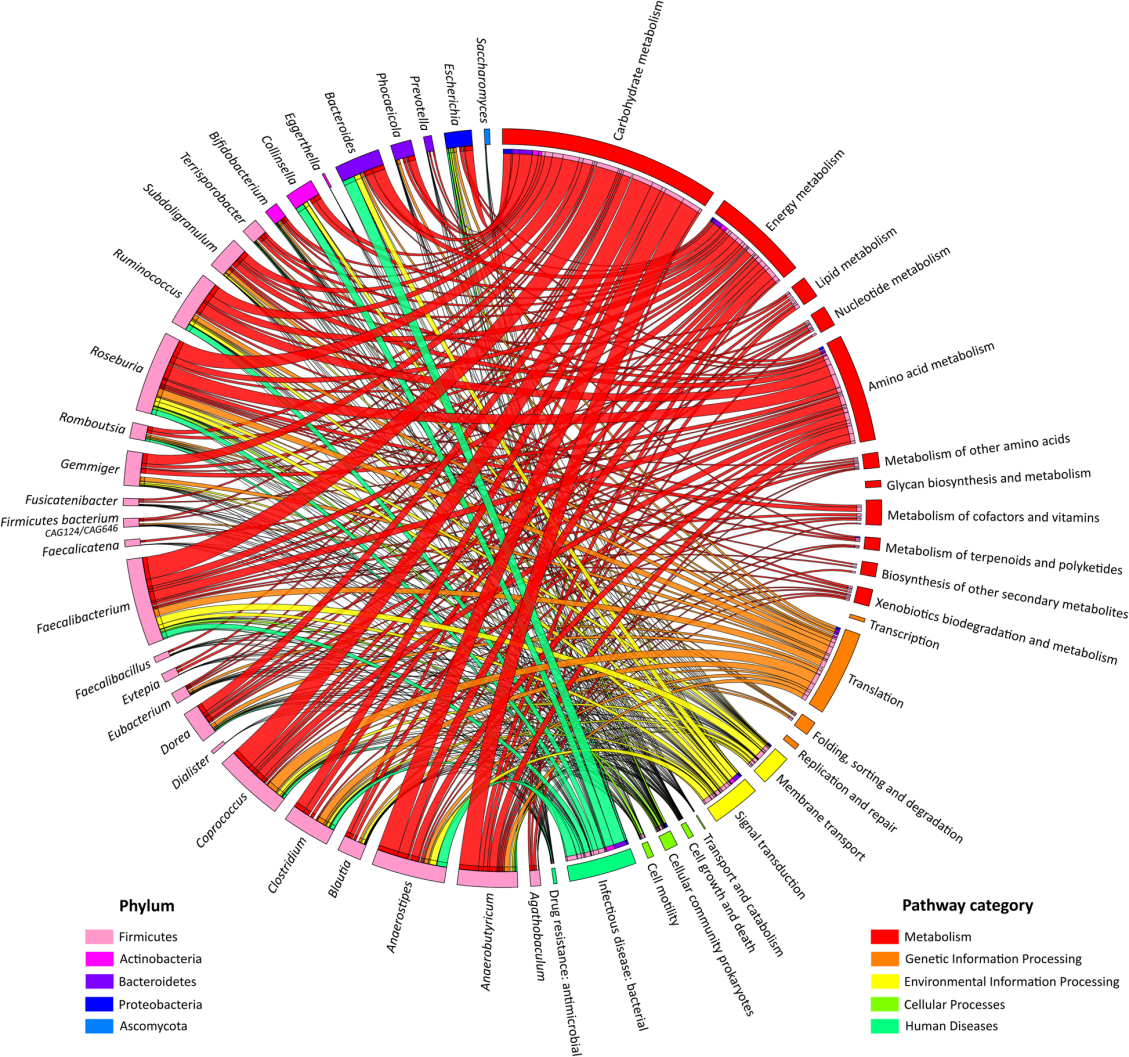
Functional pathways and genera relationships The phylum of each of the 29 microbial genera identified in the dataset is indicated as well as the functional subcategories grouped per KEGG pathway category

Notably, 71% (15,776 out of 22,250) of microbial proteins and 89% (886 out of 997) of human proteins had existing KO annotations (Table S5). Host and microbial proteins were found to be involved in 367 and 191 biological pathways, respectively, covering five functional categories: metabolism, genetic information processing, environmental information processing, cellular processes, and human diseases (Table S6). Their general distribution is shown in Fig. 4. Interestingly, the general collection of ‘carbohydrate metabolisms’ KEGG pathways has both the highest abundance rate and the highest number of KO functions (544) for the microbiota. Even pathways including less abundant proteins, such as ‘cell motility’, show a depth of coverage of over 40% (Table S6). When we delved into the contributions of different microbial genera to the functional diversity of the gut, we observed that we were able to explore functions even within relatively less represented taxa, supporting the potential of the Orbitrap Astral instrument in shedding light on the peculiar role of the entire microbiota (Fig. 5).

**Fig. 6:**
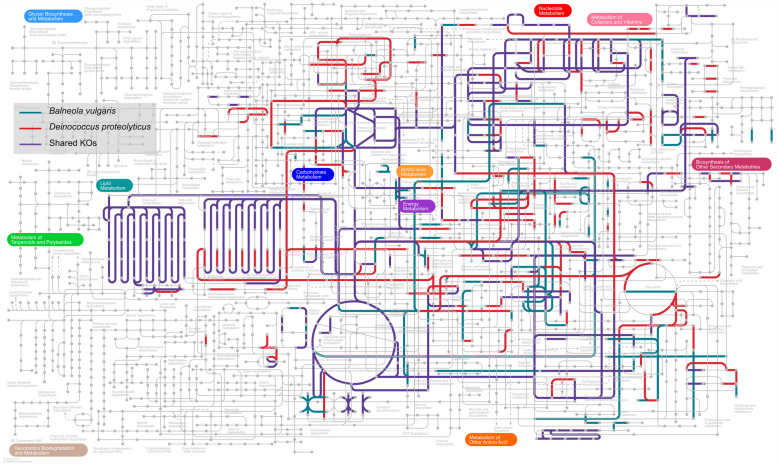
Comparative KEGG metabolism landscape of *B. vulgaris* and *D. proteolyticus*. Colors distinguish the metabolic pathways shared between the two spiked bacteria and those specific to each

Turning our attention to the spiked *B. vulgaris* and *D. proteolyticus*, annotation of their identified proteins, amounting to 743 and 1089, provided us with a functional snapshot of 65% and 62% of their proteomic profile, respectively. Figure 6 shows the overlap and specific coverage of their metabolic pathways. Although the amount of *B. vulgaris* is lower than that of *D. proteolyticus* (1:2), we found that folate cofactor production and phenylpropanoid synthesis are important within the marine bacterium. Proteins for the metabolism of several amino acids (Histidine, Tryptophane, Valine, Leucine, and Isoleucine) and proteases are specifically detected in *D. proteolyticus*, in line with the characteristics reported for this bacterium and reflected in the species epithet.

Finally, host-specific proteins were annotated within 367 KEGG-level 2 pathways, with signal transduction standing out as one of the most densely populated pathways in terms of KO annotations. The depth of coverage reached 33% for ‘Endocrine and other factor-regulated calcium reabsorption’ (Table S6). In this dataset, despite having low coverage, pathways known to play a crucial role in microbiota-host interaction, such as that related to the immune system function, are highly informative.

## Discussion

The Orbitrap Astral next-generation tandem mass spectrometer, recently released by Thermo, is based on Orbitrap technology, a new ion processor rectilinear trap developed to rapidly inject ions obtained after precursor fragmentation [5], and the powerful Astral multi-reflection analyzer. This last device incorporates multiple oscillations between two electrostatic mirrors, resulting in a total flight length for secondary ions exceeding 30 m. Its performance consists of a mass resolving power of 100,000 at the MS/MS scanning frequency of 200 Hz. The performance of this instrument has already been documented for various applications in human proteomics and for a simple mixture of peptides from three organisms, where 14,000 proteins were reported for a 28-min gradient [6]. Here, we present for the first time the results obtained for a real-life metaproteomics sample. Although the peptide mixture obtained from a human fecal sample spiked with peptides from known bacteria is very complex, the number of MS/MS scans over 60 min was over 331 thousand in DDA mode, i.e. an average of 92 scans per second. The parameters used here were optimal for short gradients, but further optimization would definitely improve results for long gradients, for which more peptide material could be used. This recorded dataset is of high quality, resulting in the identification of over 42,000 unique peptides, but even better datasets were recorded in DIA mode, with the identification of over 122,000 unique peptides in just 30 min. Information density is exceptionally high, with the size of the DDA 60 min and DIA 30 min raw files being ≈ 14 GB each. The three fold increase in unique peptides is not detrimental to the ratio of peptides per protein, indicating that the confidence in the DIA results should be in the same range as DDA results.

The approach proposed here to take advantage of the large datasets recorded by the Orbitrap Astral tandem mass spectrometer is based on (i) a DDA survey of the sample to proteotype organisms without any a priori at the genus or species level using a generic database derived from the NCBInr database, (ii) the construction of a dedicated database representing, as far as possible, the organisms that contribute the most to protein biomass, and (iii) the interpretation of large DIA datasets with a sample-specific database of limited size. Current strategies for interpreting DIA datasets based on large databases, such as metagenomic data acquired on the same sample [21] or a catalog dedicated to the gut microbiome such as MetaHit [22], face significant challenges due to the exceptionally large search space and computational limitations. The use of a peptide database gathering all peptides already identified in previous studies on the human gut microbiome is an interesting alternative that has recently been explored [23], but its a priori design may be detrimental to the characterization of atypical samples. Indeed, the two spiked bacteria used in the present study, which were never reported in previous analyses of the gut microbiome, would have been missed by conventional interpretation approaches. Here, proteotyping based on accurate and reliable taxonomic information derived from high-quality peptide sequences identifies the organisms present in the sample at genus, species, or even strain taxonomical ranks. This methodology has great potential for the rapid diagnostics of complex samples [3, 24], having the capacity to accurately estimate the biomass of each identified taxon [14]. It has been successfully applied to assess dysbiosis in the gut microbiome of COVID-19 patients [12], to identify keystone microbial players in sentinel animals [25], or to determine the presence of pathogens on ancient human remains [26, 27]. Tandem mass spectrometry proteotyping leads to a fine-grained taxonomy resolved metaproteomic strategy, as initially proposed for less complex samples [28]. Here, we constrained our proteotyping search to a subset of 100,000 MS/MS spectra due to computational limits but obtained a similar landscape of organisms to select for database construction, regardless of the Orbitrap Astral dataset employed. Consequently, a quick Orbitrap Astral DDA survey of 15–20 min gradient would in principle be more than sufficient to rapidly create the customized database for the DIA interpretation. We are convinced that this two-stage approach is appropriate for the analysis of such complex samples: (i) reliable taxonomic proteotyping is used to select the most appropriate database, and then, (ii) classical proteomics interpretation is carried out, each step being appropriately FDR-constrained. This procedure is by nature exactly the same as that used for classical proteomics: if a human or *Escherichia coli* sample is processed, the database chosen for interpretation will be selected accordingly on the basis of the prior information available. Without prior knowledge, proteotyping will easily determine whether the sample contains human or enterobacterial proteins and the final database for proteomic interpretation will logically be customized. Although the database for metaproteomic interpretation of gut microbiome samples may not be comprehensive enough to encompass all protein variant sequences present in the sample, the strategy we propose is highly effective, as demonstrated in the present study. The results of such a strategy will be further strengthened in the near future as the number of genomes available in generalist databases increases over time. Certainly, the addition of a new interpretation dimension, such as de novo sequencing interpretation [29], error-tolerant searches, or searches for multiple post-translational modifications [30], to exploit the MS/MS signals that have not yet been assigned with the current strategy, will certainly benefit the results. However, the computational limitations of the corresponding software would need to be lifted in order to be applied to the very large DIA datasets acquired by the Orbitrap Astral instrument.

DIA data can also be interpreted with an experimental spectral library based on DDA data acquired on the same sample. However, several benchmark studies have revealed that interpretation of DIA data without a library gives better results than library-based strategies [31–33] or similar results [34]. Indeed, such a spectral library for metaproteomics datasets will only be partial for interpreting DIA results as the DDA dataset is far from complete enough. Here, more specifically the parameters used for DDA acquisition in this study record more chimeric spectra than conventional parameters, thus compromising the result of the spectral library. Last, the unusual size of the dataset makes this strategy very demanding in terms of computing resources. Another alternative consists of generating a pseudo-spectral library directly from the DIA data to facilitate database construction [35], but pipelines have not been yet optimized for the Orbitrap Astral datasets. Clearly, it is of utmost importance to improve proteomic and metaproteomic software for handling giant Astral datasets and benchmark all possible strategies within the metaproteomic initiative framework [36]. The DIA interpretation results obtained here, 122,087 peptide sequences on average for a 30-min of gradient on the MetaP reference sample, can be compared favorably with results obtained very recently on similar biological material but with different instruments and parameters: 11,122 peptide sequences for a 90-min gradient [35], 49,224 peptide sequences for a 70-min gradient [34], and 70,272 peptides for a 130-min gradient [37].

The biomasses of the identified organisms estimated from the DDA and DIA datasets are relatively comparable, while the former is estimated on the number of TSMs and the latter on precursor intensity. For example, the *Balneola vulgaris* bacterium added to MetaP standard to represent 1.0% of the total peptidome was estimated at 1.0% and 1.1%, respectively. The Streptophyta (food) represented 9.9% and 8.2%, respectively. For the *Deinococcus proteolyticus* bacterium, the percentage of biomass is better measured with DIA results (3.4%) than with DDA results (5.2%). As we did not observe a significant skew in terms of biomass between the two acquisition strategies, we concluded that the signals observed with both methods are far from random among the 437,578 protein entries in the DB48 database and are reliable. In any case, further analysis of various instruments, experimental DIA parameters, and interpretation pipelines could help the metaproteomics community to adopt DIA [2, 36]. Taking into account the intensity of protein standards or specific organisms added in known quantities to the sample as monitoring indicators is relevant for such an objective.

Based on the results reported here, DIA mode appears superior to DDA mode for microbiome analysis, as more peptides and proteins can be identified and quantified, as has already been established [37–39], providing more information on the biological pathways of the system. Here, we observed that even a bacterium added at 1% in the complex fecal matrix is well covered in terms of functional characterization with a single 30-min DIA Astral analysis. However, the average of 3.0 peptides per protein group obtained in this analysis, while higher than most current metaproteomics studies, indicates that the diversity of protein sequences and the dynamic range of abundance in fecal samples are huge. Random peptide sampling by the tandem mass spectrometer therefore still occurs, even with this new generation of tandem mass spectrometers, as previously predicted [3]. As a result, further analytical efforts should be made to achieve greater coverage of this type of sample. Indeed, microbiome samples can be so complex that they present interesting challenges in terms of chromatography, mass spectrometry, and informatics. In our view, these are invaluable samples for probing and comparing the performance of next-generation tandem mass spectrometers with MS/MS acquisition frequencies above 200 Hz, which will most likely be developed and proposed in the future.

In conclusion, we report, from a single sample, the identification and quantification of 44,204 protein groups in a 90-min DIA analysis with a controlled FDR search of 1%, a groundbreaking figure compared with all reports published to date on real-life metaproteomic samples of which we are aware. This value is set to be much higher in the future, once specific optimizations have been made at all stages of the analytical procedure. The ability to encompass more than 122,000 unique peptides and 38,000 protein groups within a 30-min DIA run, while maintaining a very good repeatability across analytical runs, is also very promising. This specific record in terms of the number of peptides and proteins detected is futile in itself but allows us to glimpse the possibilities of metaproteomics for the future to tackle more complex challenges, such as consequent cohorts of samples and improved functional depth. Ultimately, the Astral mass analyzer for highly complex samples brings metaproteomics closer to routine use in clinical diagnostics.

### Supplementary Information

**Additional file 1: Table S1.** List of identified taxa by DDA proteotyping (*p* value 0.05) for six individual datasets.

**Additional file 2: Table S2.** Cumulated taxonomical results by DDA proteotyping.

**Additional file 3: Table S3.** List of organisms selected for building the DB48 protein sequence database.

**Additional file 4: Table S4.** List of identified peptides and proteins from the Orbitrap Exploris 480 mass spectrometer using a 90-min DDA acquisition.

**Additional file 5: Table S5.** List of identified proteins in the 30 min (2Da—3 ms) DIA datasets (3 replicates).

**Additional file 6: Table S6.** Functional analysis of host and microbiota proteins identified in the 30 min (2Da—3 ms) DIA datasets (3 replicates).

## Data Availability

Mass spectrometry proteomics data have been deposited to the ProteomeXchange Consortium via the PRIDE partner repository under the dataset identifiers PXD045838 (Orbitrap Astral DDA dataset), PXD046290 (15 and 30 min Orbitrap Astral DIA files), PXD046320 (60 and 90 min Orbitrap Astral DIA files), and PXD047139 (90 min Orbitrap Exploris 480  DDA files). The data are public.
